# *Il12a* Deletion Aggravates Sepsis-Induced Cardiac Dysfunction by Regulating Macrophage Polarization

**DOI:** 10.3389/fphar.2021.632912

**Published:** 2021-07-02

**Authors:** Zhen Wang, Menglin Liu, Di Ye, Jing Ye, Menglong Wang, Jianfang Liu, Yao Xu, Jishou Zhang, Mengmeng Zhao, Yongqi Feng, Shuwan Xu, Wei Pan, Zhen Luo, Dan Li, Jun Wan

**Affiliations:** ^1^ Department of Cardiology, Renmin Hospital of Wuhan University, Wuhan, China; ^2^ Cardiovascular Research Institute, Wuhan University, Wuhan, China; ^3^ Hubei Key Laboratory of Cardiology, Wuhan, China; ^4^ Department of Emergency, Renmin Hospital of Wuhan University, Wuhan, China; ^5^ Department of Pediatrics, Renmin Hospital of Wuhan University, Wuhan 430060, China

**Keywords:** sepsis, cardiac dysfunction, inflammation, macrophage, ll12a

## Abstract

Cardiac dysfunction is a well-recognized complication of sepsis and is associated with the outcome and prognosis of septic patients. Evidence suggests that *Il12a* participates in the regulation of various cardiovascular diseases, including heart failure, hypertension and acute myocardial infarction. However, the effects of *Il12a* in sepsis-induced cardiac dysfunction remain unknown. In our study, lipopolysaccharide (LPS) and cecal ligation and puncture (CLP) model were used to mimic sepsis, and cardiac *Il12a* expression was assessed. In addition, *Il12a* knockout mice were used to detect the role of *Il12a* in sepsis-related cardiac dysfunction. We observed for the first time that *Il12a* expression is upregulated in mice after LPS treatment and macrophages were the main sources of *Il12a.* In addition, our findings demonstrated that *Il12a* deletion aggravates LPS-induced cardiac dysfunction and injury, as evidenced by the increased serum and cardiac levels of lactate dehydrogenase (LDH) and cardiac creatine kinase-myocardial band (CK-MB). Moreover, *Il12a* deletion enhances LPS-induced macrophage accumulation and drives macrophages toward the M1 phenotype in LPS-treated mice. *Il12a* deletion also downregulated the activity of AMP-activated protein kinase (AMPK) but increased the phosphorylation levels of p65 (p-p65) and NF-κB inhibitor alpha (p-IκBα). In addition, *Il12a* deletion aggravates CLP-induced cardiac dysfunction and injury. Treatment with the AMPK activator AICAR abolishes the deterioration effect of *Il12a* deletion on LPS-induced cardiac dysfunction. In conclusion, *Il12a* deletion aggravated LPS-induced cardiac dysfunction and injury by exacerbating the imbalance of M1 and M2 macrophages. Our data provide evidence that *Il12a* may represent an attractive target for sepsis-induced cardiac dysfunction.

## Introduction

Sepsis is a life-threatening disease and is closely related to a high number of deaths worldwide. Clinical and epidemiological data show that 1.5 million people are infected with sepsis annually, and one-third of patients in America who die in the hospital have sepsis (Fleischmann et al., 2016; Rhee et al., 2017). Cardiac dysfunction is a well-recognized complication of sepsis and is associated with the outcome and prognosis of septic patients (Merx and Weber, 2007; van der Slikke et al., 2020). Reports indicate that approximately 60% of septic patients exhibit cardiac dysfunction, as diagnosed within the first 3 days (Vieillard-Baron, 2011). Sepsis and sepsis-related cardiac dysfunction are clearly multifactorial, including processes, such as the inflammatory response, oxidative stress injury, mitochondrial dysfunction and apoptosis (Li et al., 2019; Martin et al., 2019; Lin et al., 2020). Therefore, molecules or genes that target the above processes in sepsis and cardiac dysfunction are crucial for improved survival.

Previous studies have shown that several members of the interleukin (IL) family contribute to the pathological process of sepsis. Feng et al. reported that serum IL-6 and IL-18 concentrations are significantly increased in severe sepsis and septic shock (Feng et al., 2016). In addition, increased IL-17 and IL-27 levels are found in the serum of pediatric and adult septic patients early after sepsis onset and have been proposed as diagnostic biomarkers (Morrow et al., 2019). Weber GF et al. also reported that IL-3 potentiates inflammation and fuels a cytokine storm in sepsis, and IL-3 deficiency protects mice against sepsis (Weber et al., 2015). In addition, IL-18 deficiency attenuated lipopolysaccharide (LPS)-induced cardiac dysfunction and injury (Okuhara et al., 2017).

IL-12 is a key inflammation-related cytokine and is produced by immune cells, especially macrophages, in response to microbial pathogens (Sun et al., 2015). IL-12p35 (*Il12a*) is a common component of IL-12 and participates in the regulation of various cardiovascular diseases. Kan and his coauthor reported that *Il12a* knockout (KO) improves left anterior descending coronary artery occlusion-induced acute myocardial infarction by promoting anti-inflammatory functions of monocytes (Kan et al., 2016). In addition, our previous studies showed that *Il12a* deficiency aggravated acute cardiac injury by amplifying the inflammatory response and oxidative stress in doxorubicin-treated mice (Ye et al., 2018). However, whether *Il12a* plays a role in sepsis and sepsis-related cardiac dysfunction is unknown. In the current study, we determined the function of *Il12a* in sepsis-related cardiac dysfunction and elucidated its underlying mechanisms.

## Materials and Methods

### Animals and Experimental Model

*Il12a* knockout (*Il12a*-KO) mice with a C57BL/6 background were purchased from the Institute of Model Zoology, Nanjing University (imported from the Jackson Laboratory), and wild type (WT) mice in the same brood were used as controls (Ye et al., 2018). All mice were housed in environmentally controlled and well-ventilated cages with free access to standard food and water. Eight-to ten-week-old male *Il12a* KO mice and their WT littermates were randomly assigned into the following groups: sham + WT group (n = 10), sham + KO group (n = 10), LPS + WT group (n = 10) and LPS + KO group (n = 10). The sepsis-related cardiac dysfunction mouse model was induced by LPS injection intraperitoneally (10 mg/kg, Sigma-Aldrich, United States), as described in previous studies (Preau et al., 2016; Li et al., 2019). The sham groups were given an intraperitoneal injection with an isovolumetric dose of saline. Six hours later, mice were anesthetized and transthoracic echocardiography was performed to determine their cardiac function. Then, mice were sacrificed, and the myocardial tissues were obtained for further measurement. In addition, polymicrobial sepsis was created by cecal ligation and puncture (CLP), as described in a previous study (Lv et al., 2018). The study was approved by the Animal Care and Use Committee of Renmin Hospital of Wuhan University and performed in accordance with the NIH Guidelines for the Care and Use of Laboratory Animals.

### Echocardiography and Hemodynamic Analysis

Cardiac function was evaluated by echocardiography and hemodynamic analysis as described previously (Wang et al., 2018). In brief, transthoracic echocardiography was performed using a Mylab30CV ultrasound (Biosound Esaote), and data on cardiac function parameters, including left ventricular end-diastolic diameter and end-systolic diameter (LVEDd and LVESd), ejection fraction (LVEF) and fractional shortening (LVFS), were collected. In addition, hemodynamics were measured in mice using a Millar Pressure-Volume System (Millar, Inc., United States).

### Survival Analysis

The mice in each group were intraperitoneally injected with 25 mg/kg LPS or received CLP for survival analysis, as described in a previous study (Jia et al., 2018). The number of deaths and percent survival were observed and calculated every 12 h for 5 days after LPS injection or CLP.

### 2 Cell Culture

Bone marrow-derived macrophages (BMDMs) were isolated from WT or *Il12a* KO mice and cultured in complete DMEM supplemented with murine macrophage colony-stimulating factor (50 ng/ml), as described in our previous study (Wang et al., 2018). BMDMs were treated with LPS (1 μg/ml) or 5-aminoimidazole-4-carboxamide 1-β-d-ribofuranoside (AICAR, 500 μM) for 24 h to extract cellular RNA for mRNA analysis.

### Biochemical Determination

Blood specimens were obtained and centrifugated at 4,000 × g at 4°C for 15 min (Ye et al., 2020). Thereafter, serum specimens were collected and stored in a −80°C freezer until subsequent analyses. Additionally, cardiac tissues were homogenized in phosphate-buffered saline. Serum and cardiac tissue concentrations of creatine kinase-myocardial band (CK-MB, Jiancheng Bioengineer, China) and lactate dehydrogenase (LDH, Jiancheng Bioengineer, China) were detected according to the manufacturer's instructions.

### Histological Analysis

Heart specimens were obtained immediately after sacrifice and fixed in 10% formalin for 48 h. Thereafter, the specimens were dehydrated and embedded in paraffin and then cut transversely into 5-μm sections. For immunohistochemistry, the sections were blocked with 10% BSA and incubated with primary antibodies against CD68 (1:100, Abcam, United States) overnight at 4°C. Then, the sections were incubated with anti-rabbit HRP reagent (Gene Tech, Shanghai, China) and DAB (Gene Tech, Shanghai, China). For immunofluorescence, the sections were incubated with primary antibodies against CD68 (1:200, Abcam, United States), CD206 (1:200, R&D Systems, United States) and CD80 (1:200, R&D Systems, United States) overnight at 4°C. In addition, terminal deoxynucleotidyl transferase-mediated dUTP nick end labeling (TUNEL) staining was conducted using commercially available apoptosis detection kits (Roche, Basel, Switzerland). All images were obtained with a light or fluorescence microscope and analyzed using Image-Pro Plus6.0 software.

### Quantitative Real-Time RT-PCR

Total RNA was collected using TRIzol reagent and reverse transcribed to cDNA according to a previous protocol (Ye et al., 2020). Quantitative real-time RT-PCR was performed using LightCycler 480 (Roche, Switzerland) according to the manufacturer's recommendation. The relative expression of target genes was normalized to that of glyceraldehyde-3-phosphate dehydrogenase (GAPDH). All primer sequences in our study are listed in Table 1.

**TABLE 1:** Primers for quantitative polymerase chain reaction.

Gene	Forward primer (5′-3′)	Reverse primer (5′-3′)
IL-12p35	AGT​TTG​GCC​AGG​GTC​ATT​CC	TCT​CTG​GCC​GTC​TTC​ACC​AT
IL-1β	GGG​CCT​CAA​AGG​AAA​GAA​TC	TAC​CAG​TTG​GGG​AAC​TCT​GC
IL-6	AGT​TGC​CTT​CTT​GGG​ACT​GA	TCC​ACG​ATT​TCC​CAG​AGA​AC
TNF-α	CCC​AGG​GAC​CTC​TCT​CTA​ATC	ATG​GGC​TAC​AGG​CTT​GTC​ACT
CD80	GGC​CTG​AAG​AAG​CAT​TAG​CTG	GAG​GCT​TCA​CCT​AGA​GAA​CCG
CD86	GCT​TCA​GTT​ACT​GTG​GCC​CT	TGT​CAG​CGT​TAC​TAT​CCC​GC
CD163	TCC​ACA​CGT​CCA​GAA​CAG​TC	CCT​TGG​AAA​CAG​AGA​CAG​GC
CD206	CAG​GTG​TGG​GCT​CAG​GTA​GT	TGT​GGT​GAG​CTG​AAA​GGT​GA
iNOS	CGA​AAC​GCT​TCA​CTT​CCA​A	TGA​GCC​TAT​ATT​GCT​GTG​GCT
Arg-1	AAC​ACG​GCA​GTG​GCT​TTA​ACC	GGT​TTT​CAT​GTG​GCG​CAT​TC
GAPDH	ACT​CCA​CTC​ACG​GCA​AAT​TC	TCT​CCA​TGG​TGG​TGA​AGA​CA

### Western Blot Analysis

Total protein was extracted from LV tissues using RIPA buffer and quantified using a BCA protein assay kit (Thermo Fisher Scientific, USA), as described previously (Wang et al., 2019). Then, proteins (50 μg per sample) were separated by SDS-PAGE gel and transferred to PVDF membranes (Millipore, Beijing, China). The membranes were incubated with primary antibodies overnight at 4 °C, including anti-*Il12a* (1:1,000, Abcam, United States), anti-Bcl-2 (1:1,000, Abcam,United States, anti-Bax (1:1,000, CST, United States), anti-c-caspase3 (1:1,000, CST, United States), anti-*p*-AMPKα (1:1,000, CST, United States), anti-AMPKα (1:1,000, CST, United States), anti-p-p65 (1:1,000, Abcam, United States), anti-p65 (1:1,000, CST, United States), anti-*p*-IκBα (1:1,000, CST, United States), anti-IκBα (1:1,000, CST, United States), and anti-GAPDH (1:1,000, CST, United States. Then, the membranes were incubated with secondary antibodies and visualized using an Odyssey infrared imaging system (LI-COR, United States). The levels of protein expression were normalized to that of GAPDH.

### Statistics

All the values are presented as the means ± standard deviations (SD). Differences were assessed with Student's t test or one-way analysis of variance (ANOVA) as appropriate. *p*-values of less than 0.05 were considered significant.

## Results

### *Il12a* Expression is Upregulated in Mice After LPS Treatment

**FIGURE 1:**
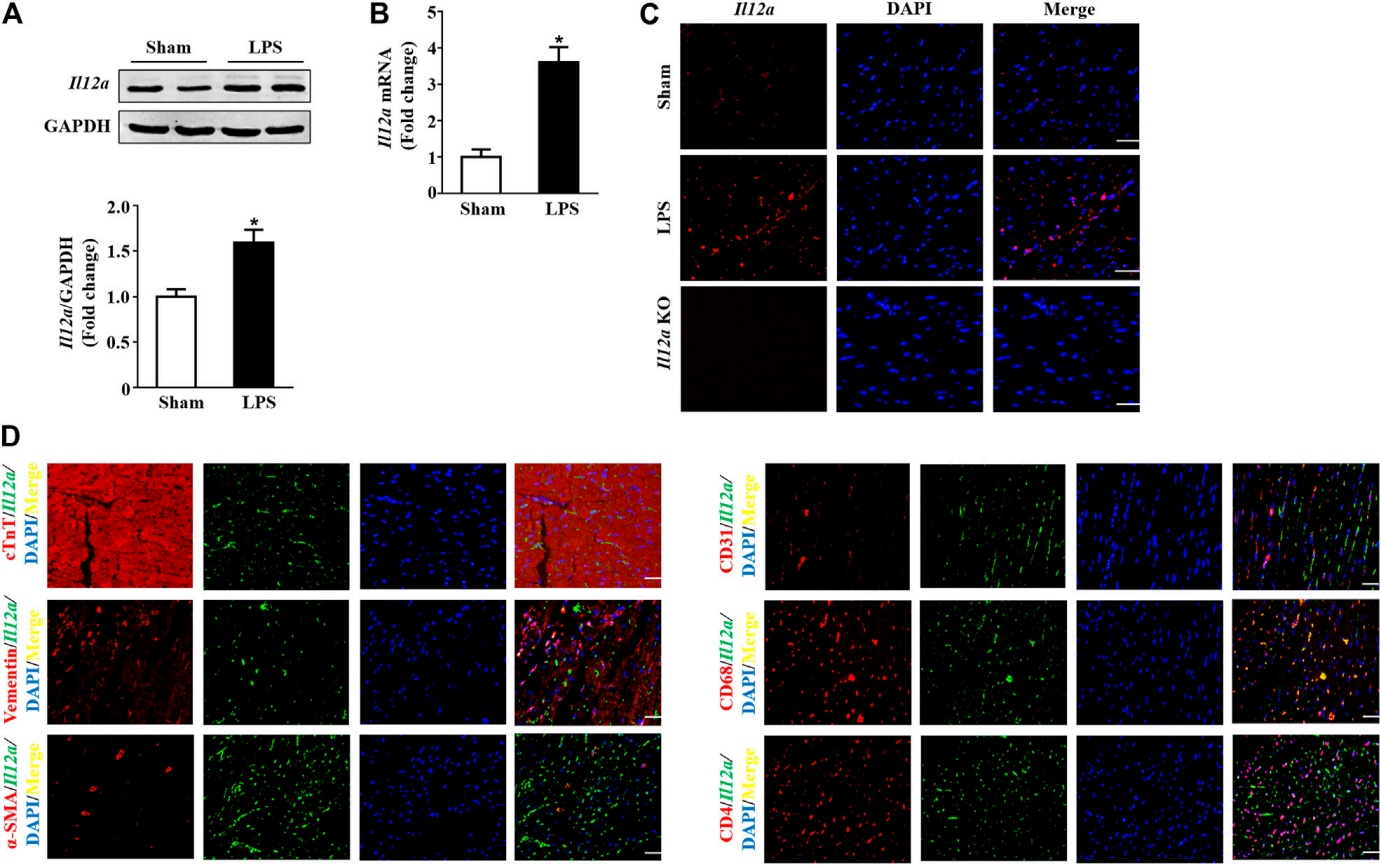
*Il12a* expression is upregulated in mice after LPS treatment. **(A,B)** Western blot and RT-PCR analysis of *Il12a* protein and mRNA expression levels in the hearts of each group (n = 4). **(C)** Immunofluorescence staining for *Il12a* in the hearts of each group (n = 4; scale bar, 50 μm). **(D)** The source of *Il12a* in the heart of mice after LPS treatment was detected by double immunofluorescence staining (n = 4; scale bar, 50 μm). *p < 0.05 compared with the sham group.

To investigate the function of *Il12a* in sepsis-related cardiac dysfunction, we measured the expression of *Il12a* in the hearts of LPS-treated mice. The results showed that *Il12a* expression was upregulated in the hearts of LPS-treated mice compared with control mice (Figures 1A–C). In addition, double immunofluorescence staining showed that macrophages were the main sources of *Il12a* (Figure 1D).

### *Il12a* Deletion Aggravates LPS-Induced Cardiac Injury

**FIGURE 2:**
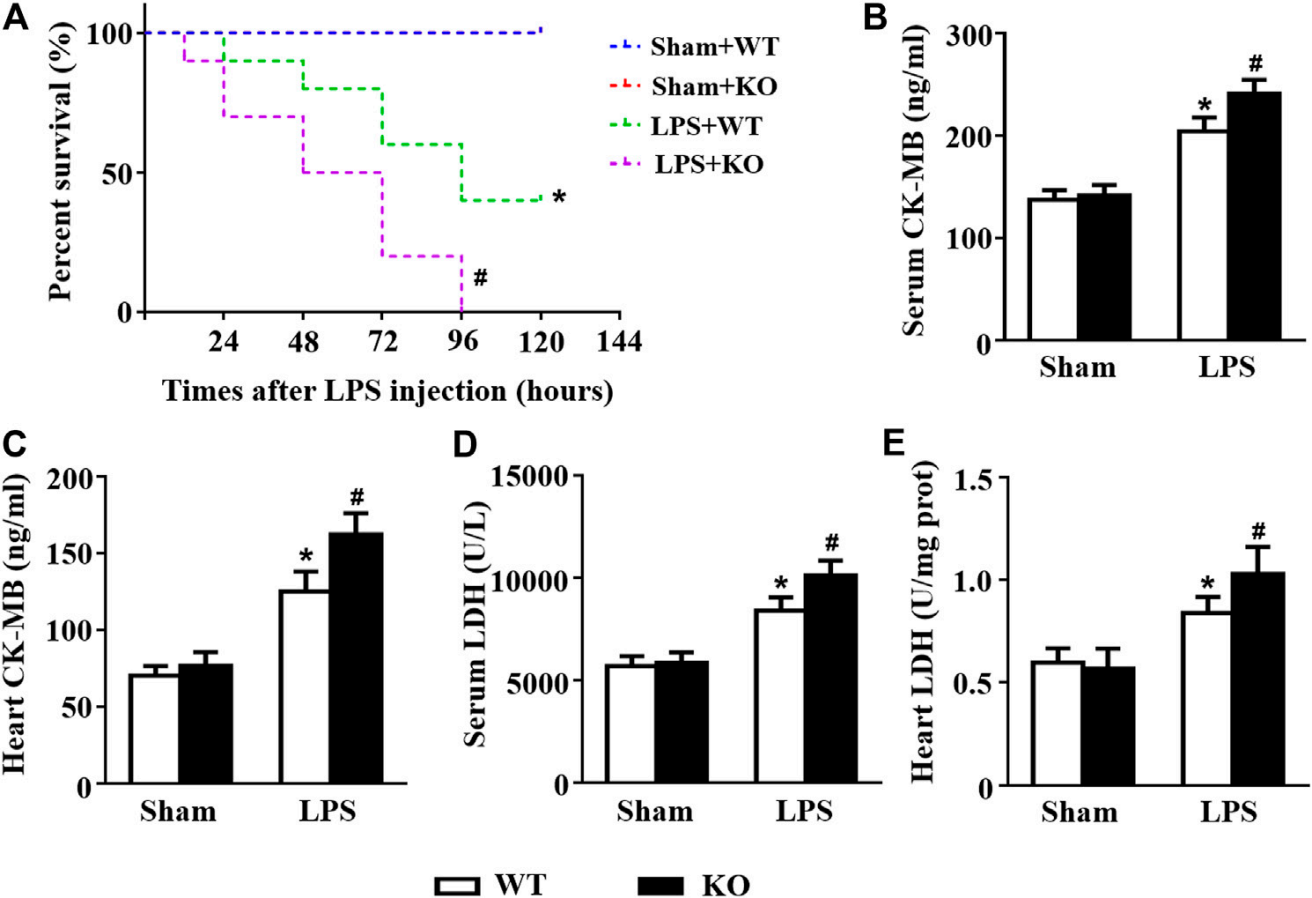
*Il12a* deletion aggravates LPS-induced cardiac injury. **(A)** Effect of *Il12a* deficiency on the survival rate after LPS treatment (n = 10). **(B,C)** CK-MB and LDH serum levels were assessed in each group (n = 4). **(D,E)** The cardiac levels of CK-MB and LDH were measured in each group (n = 4). *p < 0.05 compared with the sham + WT group; ^#^ *p* < 0.05 compared with the LPS + WT group.

Subsequently, we observed the effects of *Il12a* deletion on the survival and cardiac injury of LPS-treated mice. LPS treatment induced a decrease in the survival rate, and *Il12a* deficiency further decreased the survival rate of mice (Figure 2A). LDH and CK-MB are sensitive biomarkers for myocardial injury. Thus, we measured LDH and CK-MB levels in mice. The results showed that LPS stimulation dramatically increased LDH and CK-MB levels in both the serum and heart (Figures 2B–E). Compared to LPS treatment alone, *Il12a* deficiency further increased LDH and CK-MB levels, indicating that *Il12a* deficiency aggravates LPS-induced cardiac injury (Figures 2B–E).

### *Il12a* Deletion Exacerbates LPS-Induced Cardiac Dysfunction

**FIGURE 3:**
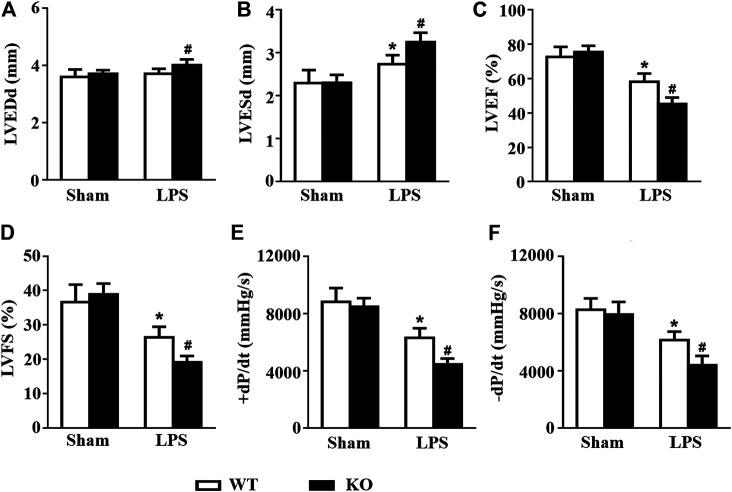
*Il12a* deletion exacerbates LPS-induced cardiac dysfunction. **(A–D)** Echocardiographic analysis of LVEDd, LVESd, LVEF and LVFS in each group (n = 8). **(D–E)** Hemodynamic analysis of +dP/dt and -dP/dt in each group **(n = 6)**. *p < 0.05 compared with the sham + WT group; ^#^ *p* < 0.05 compared with the LPS + WT group.

Consistent with the results of previous studies, LPS stimulation induced obvious cardiac dysfunction in mice, as indicated by the increased LVESd and decreased LVEF and LVFS. However, *Il12a* deficiency further exacerbates LPS-induced cardiac dysfunction (Figures 3A–D). Invasive hemodynamic results also showed that LPS-induced LV systolic and diastolic dysfunction was further increased by *Il12a* deletion (Figures 3E–H).

### *Il12a* Deletion Exacerbates Macrophage Accumulation in the Heart

**FIGURE 4:**
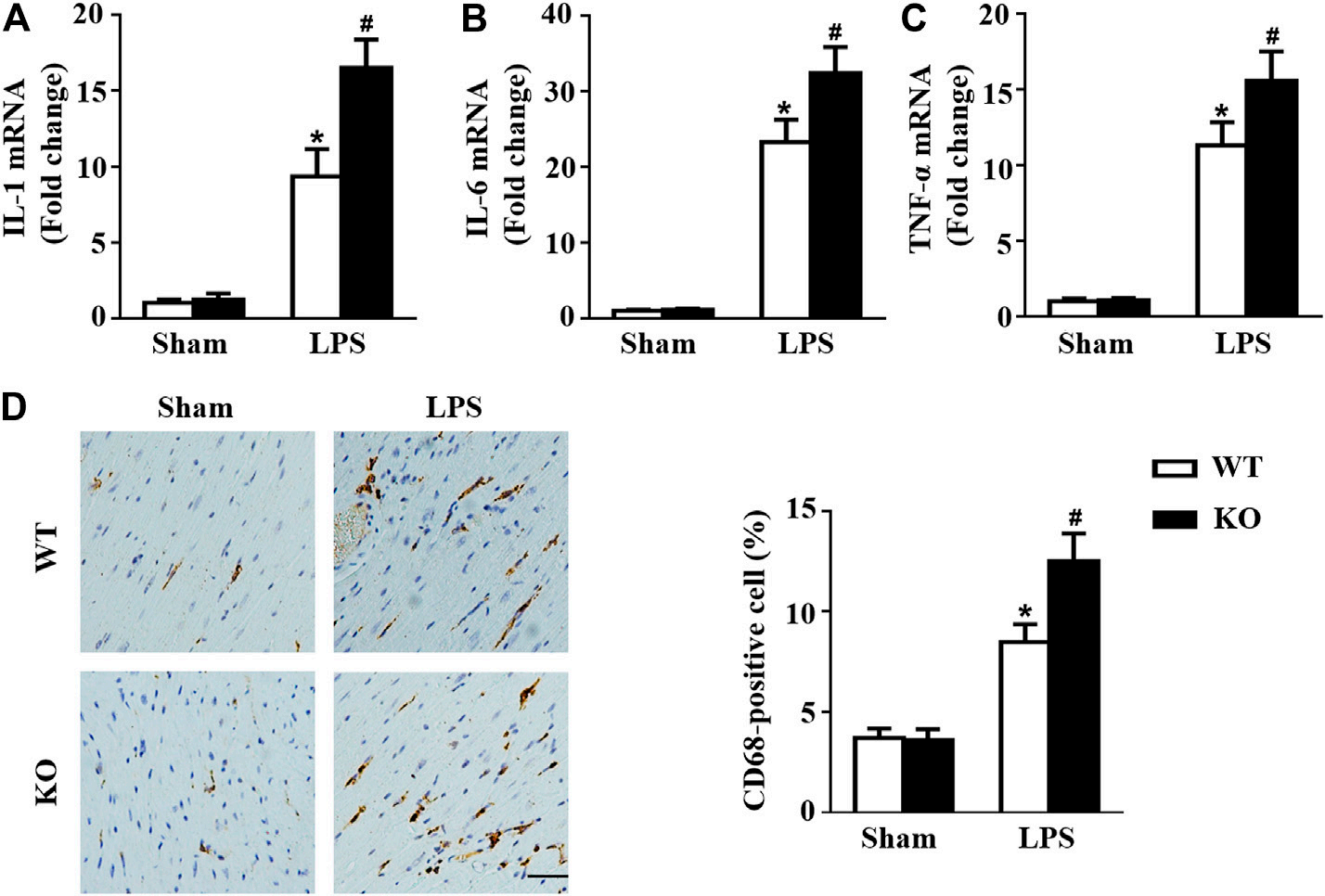
*Il12a* deletion mediates macrophage polarization in the heart. **(A–C)** RT-PCR analysis of IL-1β, IL-6 and TNF-α mRNA expression in each group (n = 4). **(D)** Immunohistochemical analysis of CD68 in heart sections (n = 4; scale bar, 50 μm). *p < 0.05 compared with the sham + WT group; ^#^ *p* < 0.05 compared with the LPS + WT group.

Macrophages are important immune cells and are involved in the progression of sepsis (Kumar, 2018; Karakike and Giamarellos-Bourboulis, 2019). Thus, we observed the effect of *Il12a* deletion on macrophage infiltration in mice. Consistent with the results of previous studies, mice subjected to LPS treatment exhibited significant upregulation of cytokine secretion by macrophages, including secretion of IL-1, IL-6 and tumor necrosis factor-α (TNF-α) (Figures 4A–C). However, *Il12a* deficiency further increases the expression of those cytokines (Figures 4A–C). Similarly, the immunohistochemistry results showed that LPS-induced macrophage accumulation in the heart was further increased by *Il12a* deletion (Figure 4D).

### *Il12a* Deletion Mediates Macrophage Polarization in the Heart

**FIGURE 5:**
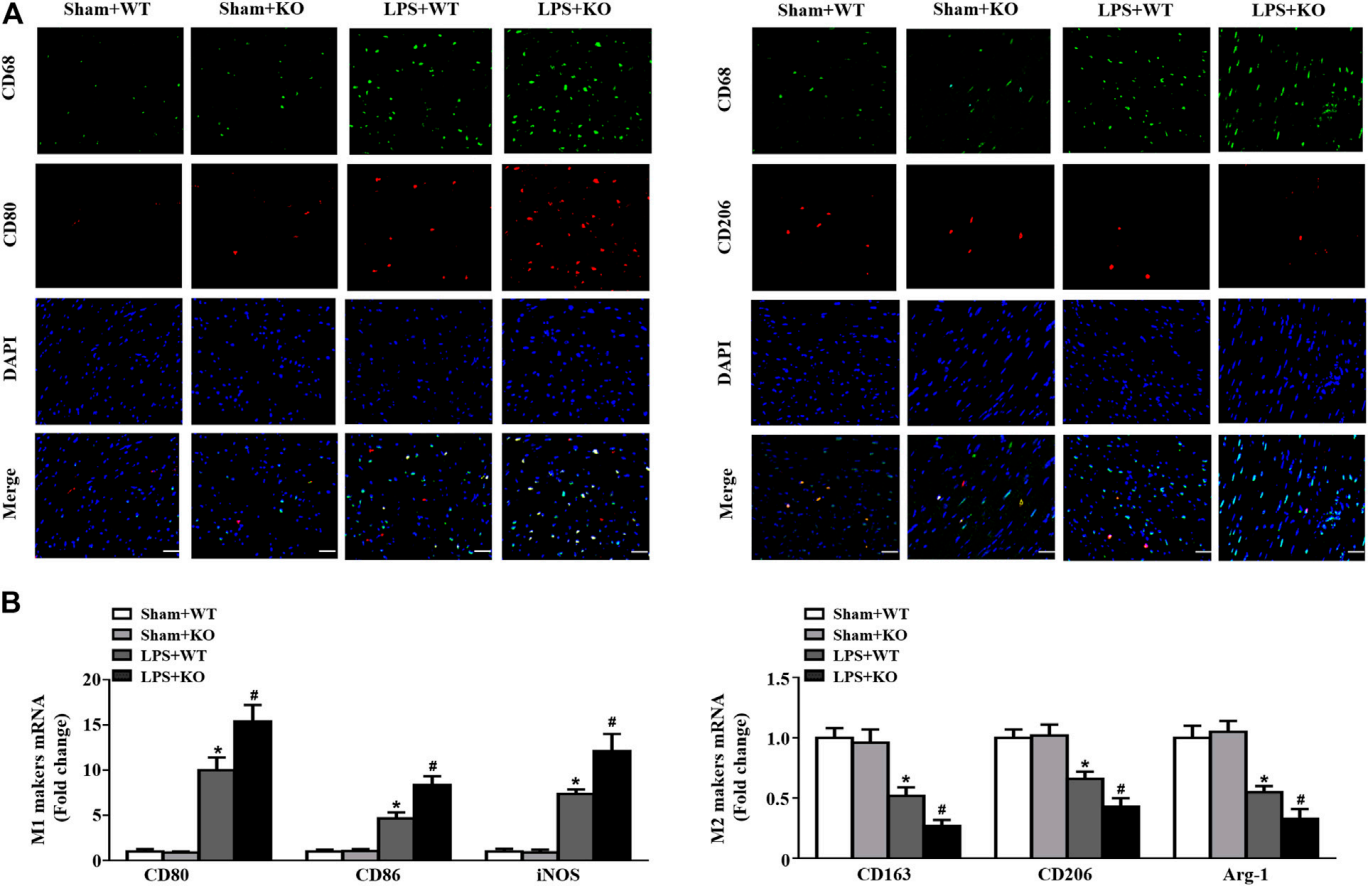
*Il12a* deletion mediates macrophage polarization in the heart. **(A)** Immunofluorescence analysis of CD80 and CD206 in the hearts of each group (n = 4; scale bar, 50 μm) **(B)** RT-PCR analysis of CD80, CD86, iNOS, CD163, CD206 and Arg-1 mRNA expression in hearts of each group (n = 4). *p < 0.05 compared with the sham + WT group; ^#^ *p* < 0.05 compared with the LPS + WT group.

We further analyzed the effect of *Il12a* deletion on macrophage differentiation in the heart. Immunofluorescence analysis demonstrated obvious infiltration of M1 macrophages (CD68^+^/CD80^+^), and M2 macrophages (CD68^+^/CD206^+^) were markedly decreased in the LPS group compared with the Sham group. *Il12a* deletion further increased the recruitment of M1 macrophages and reduced the number of M2 macrophages after LPS stimulation. In addition, similar results were observed using RT-PCR, which demonstrated that *Il12a* deletion further increased CD80, CD86 and inducible nitric oxide synthase (iNOS) mRNA levels and decreased CD163, CD206 and Arg-1 mRNA levels (Figure 5B). Therefore, *Il12a* deficiency exacerbates LPS-induced cardiac dysfunction by driving macrophages toward the M1 phenotype.

### *Il12a* Deletion Aggravates LPS-Induced Myocardial Apoptosis

**FIGURE 6:**
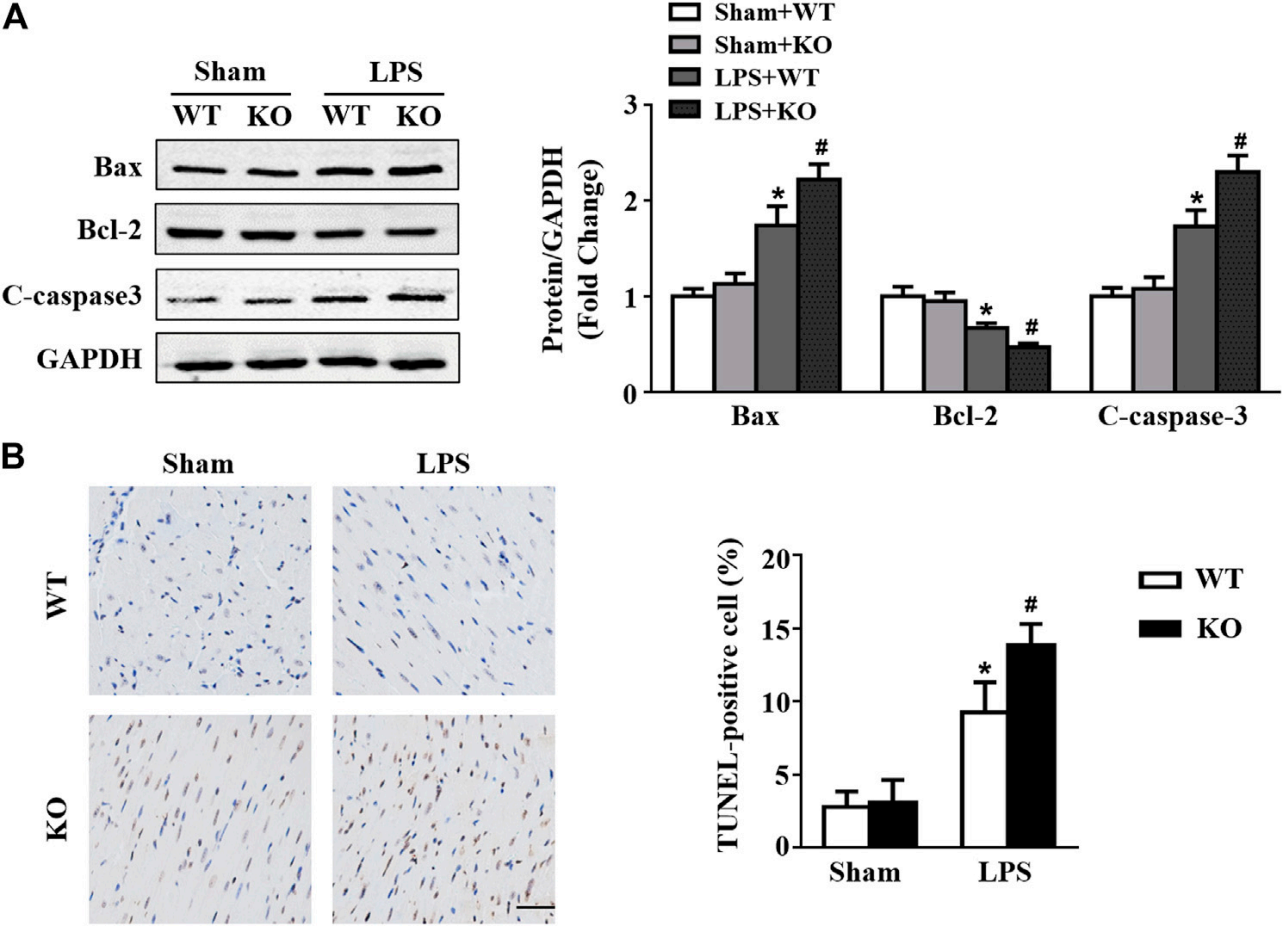
*Il12a* deletion aggravates LPS-induced myocardial apoptosis. **(A)** Western blot analysis of Bax, Bcl-2 and c-caspase-3 protein in each group (n = 4). **(C)** TUNEL staining and the quantitative results of heart tissues in each group (n = 4; scale bar, 50 μm). *p < 0.05 compared with the sham + WT group; #*p* < 0.05 compared with the LPS + WT group.

Western blot results showed that LPS stimulation increased Bax and c-caspase3 levels and decreased Bcl2 levels in hearts (Figure 6A). In addition, *Il12a* deficiency further increased Bax and c-caspase3 expression and decreased Bcl2 expression in hearts after LPS treatment (Figure 6A). Compared with the control group, the number of TUNEL-positive cells in hearts was significantly increased in the LPS group and further increased by *Il12a* deletion (Figure 6B).

### *Il12a* Deletion Mediates Macrophage Polarization via the AMP-Activated Protein Kinase (AMPK)/NF-κB pathway

**FIGURE 7:**
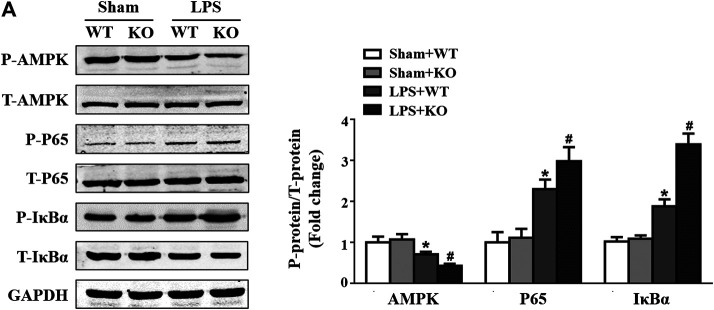
*Il12a* deletion mediates macrophage polarization via the AMPK/NF-κB pathway. **(A)** Western blot analysis of *p*-AMPK, AMPK, p-p65, p65, *p*-IκBα and IκBα protein in each group (n = 4). *p < 0.05 compared with the sham + WT group; ^#^ *p* < 0.05 compared with the LPS + WT group.

A previous study showed that the AMPK/NF-κB signaling pathway participates in the regulation of macrophage polarization (Kim et al., 2019; Liu et al., 2019); thus, we evaluated the effect of *Il12a* deletion on the AMPK/NF-κB signaling pathway. Consistent with the results of previous studies, LPS stimulation significantly decreased AMPKα phosphorylation and increased p65 and NF-κB inhibitor alpha (IκBα) phosphorylation. However, *Il12a* deficiency further decreases AMPKα phosphorylation and increases p65 and IκBα phosphorylation (Figures 7A,B).

### *Il12a* Deletion Aggravates CLP-Induced Cardiac Injury

**FIGURE 8:**
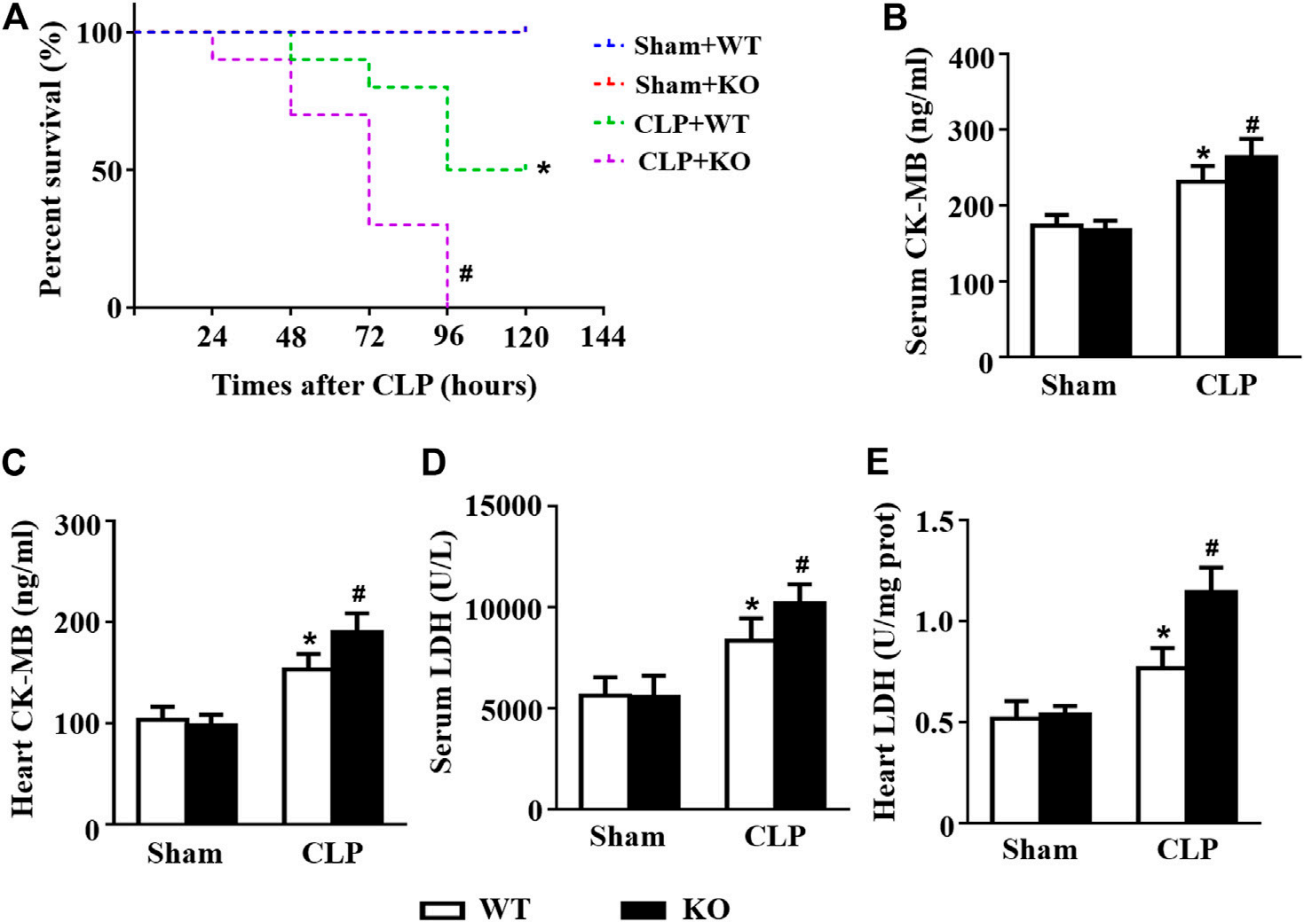
*Il12a* deletion aggravates CLP-induced cardiac injury. **(A)** Effect of *Il12a* deficiency on the survival rate after CLP treatment (n = 10). **(B–C)** CK-MB and LDH serum levels were assessed in each group (n = 4). **(D–E)** The cardiac levels of CK-MB and LDH were measured in each group (n = 4). *p < 0.05 compared with the sham + WT group; ^#^ *p* < 0.05 compared with the CLP + WT group.

The CLP model was used to further confirm the protective effect of *Il12a* in sepsis, and the results showed that *Il12a* deficiency further decreased the survival rate of mice after CLP challenge (Figure 8A). In addition, *Il12a* deficiency further increased LDH and CK-MB levels in both the serum and heart, indicating that *Il12a* deficiency aggravates CLP-induced cardiac injury (Figures 8B–E).

### *Il12a* Deletion Exacerbates CLP-Induced Cardiac Dysfunction

**FIGURE 9:**
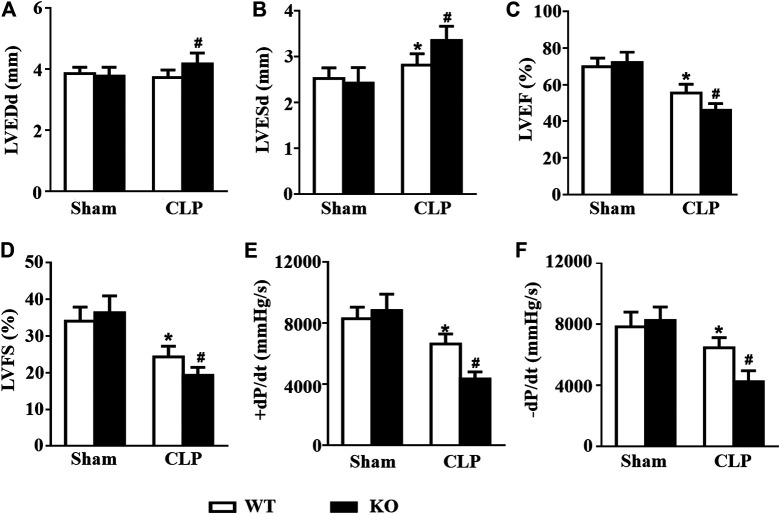
*Il12a* deletion exacerbates CLP-induced cardiac dysfunction. **(A–D)** Echocardiographic analysis of LVEDd, LVESd, LVEF and LVFS in each group (n = 8). **(D,E)** Hemodynamic analysis of +dP/dt and -dP/dt in each group (n = 6). *p < 0.05 compared with the sham + WT group; ^#^ *p* < 0.05 compared with the CLP + WT group.

Consistent with the results of previous studies, CLP challenge induced obvious cardiac dysfunction in mice, as indicated by the increased LVESd and decreased LVEF and LVFS. However, *Il12a* deficiency further exacerbates LPS-induced cardiac dysfunction (Figures 9A–D). Invasive hemodynamic results also showed that CLP-induced LV systolic and diastolic dysfunction was further increased by *Il12a* deletion (Figures 9E–H).

### AMPK Activation Abolishes the Deterioration Effect of *Il12a* Deletion on LPS-Induced Cardiac Dysfunction

**FIGURE 10:**
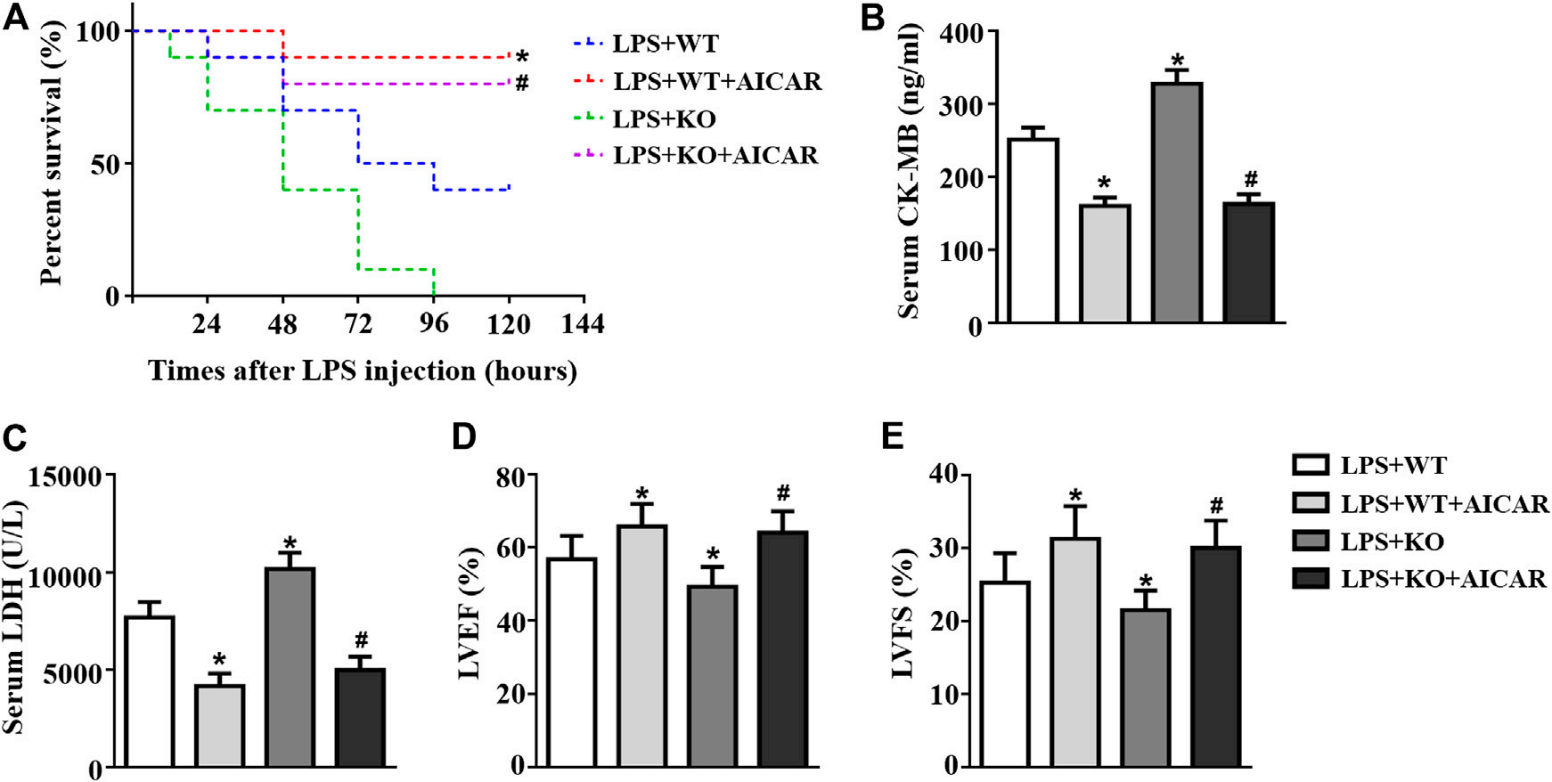
AMPK activation abolishes the deterioration effect of *Il12a* deletion on LPS-induced cardiac dysfunction. **(A)** Effect of *Il12a* deficiency on the survival rate after CLP treatment (n = 10). **(B,C)** CK-MB and LDH serum levels were assessed in each group (n = 4). **(D,E)** Echocardiographic analysis of LVEF and LVFS in each group (n = 4). *p < 0.05 compared with the LPS + WT group; ^#^ *p* < 0.05 compared with the LPS + KO group.

We further analyzed the effect of AMPK activation on *Il12a* deficiency in the heart. The results showed that treatment with the AMPK activator AICAR increased the survival rate in *Il12a* deletion mice after LPS challenge (Figure 10A). In addition, the effect of *Il12a* deletion on LPS-induced cardiac dysfunction and injury was prevented by AICAR (Figures 10B–E).

### AMPK Activation Abolishes *Il12a* Deletion-Mediated M1 Macrophage Polarization

**FIGURE 11:**
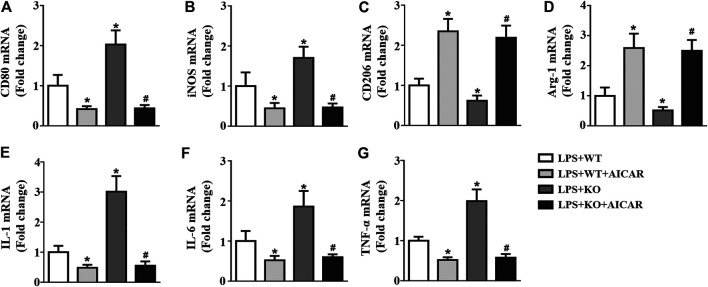
AMPK activation abolishes *Il12a* deletion mediated M1 macrophage polarization RT-PCR analysis of CD80 **(A)**, iNOS **(B)**, CD206 **(C)**, Arg-1 **(D)**, IL-1 **(E)**, IL-6 **(F)** and TNF-α **(G)** mRNA expression in each group (n = 4). *p < 0.05 compared with the LPS + WT group; ^#^ *p* < 0.05 compared with the LPS + KO group.

We further analyzed the effect of AMPK activation in macrophage polarization. The results showed that *Il12a* deletion promotes M1 macrophage polarization and decreases M2 macrophage polarization (Figures 11A–D). *Il12a* deletion also increased the expression levels of the macrophage-secreted proinflammatory cytokines, including IL-1, IL-6 and TNF-α (Figures 11E–G). However, AICAR treatment abolishes *Il12a* deletion mediated M1 macrophage polarization and decreases inflammatory cytokine production *in vitro* (Figures 11A–G).

## Discussion

In the present study, we clearly revealed the function of *Il12a* in sepsis-related cardiac dysfunction and elucidated its underlying mechanisms. We observed for the first time that *Il12a* expression is upregulated in mice after LPS treatment and that macrophages were the main sources of *Il12a*. In addition, our findings demonstrated that *Il12a* deletion decreased the survival rate and exacerbated cardiac dysfunction and myocardial injury in LPS and CLP-treated mice. We also observed that *Il12a* deletion enhances LPS-induced macrophage accumulation and drives macrophages toward the M1 phenotype. Furthermore, we noticed that *Il12a* deletion-mediated macrophage polarization is related to the AMPK/NF-κB pathway.

Sepsis-related cardiac dysfunction is a well-recognized complication of sepsis and is associated with the outcome and prognosis of septic patients. Clinical studies show that the 28-days mortality rate in hospitalized sepsis patients is between 16 and 47% in the presence of myocardial dysfunction (Charpentier et al., 2004; Durand et al., 2017). Many clinical and animal experimental studies have explored the pathophysiology and potential mechanisms of myocardial dysfunction in sepsis; however, these processes are not completely understood. Recently, growing evidence has indicated that the inflammatory cascade is central among the mechanisms of sepsis-related cardiac dysfunction. In particular, multiple inflammatory cytokines, including ILs, TNF-α and interferons, are implicated in the development of sepsis (Rackov et al., 2017; Lendak et al., 2018). These proinflammatory and anti-inflammatory mediators mediate the inflammatory cascade defense and are involved in the pathogenesis of sepsis. Undoubtedly, promotion or inhibition of the release of these proinflammatory and anti-inflammatory cytokines can be very effective in improving the prognosis of sepsis and sepsis-related cardiac dysfunction.

Evidence suggests that *Il12a* participates in the regulation of various cardiovascular diseases and exhibits very different biological activities, including pro-inflammatory and anti-inflammatory activities (Li et al., 2012; Kan et al., 2016; Huang et al., 2019; Ye et al., 2019). In an atherosclerosis model induced by a high-fat diet, *Il12a* deletion ameliorated atherosclerotic lesion formation and macrophage infiltration in apolipoprotein E (ApoE) KO mice (Huang et al., 2019). Kan et al. reported that *Il12a* deletion improves left anterior descending coronary artery occlusion-induced acute myocardial infarction by promoting anti-inflammatory functions of monocytes (Kan et al., 2016). In contrast, Li et al. reported that *Il12a* deletion aggravated angiotensin II-induced cardiac inflammation and fibrosis. Our previous studies also showed that *Il12a* deficiency aggravates vascular dysfunction and promotes blood pressure elevation in a hypertensive mouse model (Ye et al., 2019). In this study, we first measured Il12a expression in the hearts of LPS-treated mice, and the results showed that Il12a expression was upregulated in the hearts of mice after LPS challenge. In addition, *Il12a* deletion decreased the survival rate and exacerbated cardiac dysfunction and myocardial injury in LPS and CLP-treated mice. Thus, we hypothesize that the regulatory roles of *Il12a* in the inflammatory response are related to different inflammatory microenvironments.

Macrophages are important immune cells in the inflammatory process and immune homeostasis and have been reported to be a critical factor in the progression of sepsis and sepsis-related myocardial injury (Kumar, 2018; Ardura et al., 2019; Karakike & Giamarellos-Bourboulis, 2019). Activated macrophages can differentiate into M1 or M2 phenotypes. M1 macrophages show a proinflammatory phenotype associated with inflammation and tissue injury, whereas M2 macrophages are involved in tissue repair and angiogenesis (Liu et al., 2014; Sica et al., 2015). In the early stage of sepsis, macrophages may be excessively activated and undergo M1 differentiation, resulting in the production of excessive proinflammatory cytokines, which has been identified as a major cause of the high mortality rate of sepsis (Angus and van der Poll, 2013; Cheng et al., 2018). Thus, regulating the balance of M1 and M2 macrophages is an attractive approach for attenuating sepsis-induced cardiac injury (Feng et al., 2014; Li et al., 2018; Zhang et al., 2020). In this study, we first assessed the source of *Il12a* in the hearts of LPS-treated mice, and the results showed that macrophages were the main sources of *Il12a,* which is consistent with the results of a previous study (Li et al., 2012)*. Il12a* deletion significantly exacerbates macrophage accumulation and increased the expression levels of macrophage-secreted proinflammatory cytokines. In addition, our data indicated that *Il12a* deletion promoted M1 macrophage differentiation but inhibited M2 macrophage differentiation. These results suggested that the deterioration of *Il12a* deletion in sepsis-related cardiac dysfunction is related to the imbalance of M1 and M2 macrophages. However, the specific mechanism by which *Il12a* regulates macrophage differentiation needs to be further explored.

AMPK is a serine/threonine protein kinase that regulates cellular metabolic stress and energy homeostasis and is involved in determining the macrophage phenotype (O’Neill and Hardie, 2013; Wu et al., 2019). AMPK facilitates the development of an anti-inflammatory state (i.e., M2 macrophage polarization), and stimulation of macrophages with LPS decreases AMPK activation (Sag et al., 2008; Ye et al., 2020). Research has also shown that inhibition of AMPK activity induces the M1 macrophage phenotype and increases the release of M1 macrophage-associated cytokines in sepsis (Kumar, 2018; Wang et al., 2018). NF-κB is a central transcription factor, and its associated signaling pathway is important in inflammation and can be negatively affected by AMPK in macrophages (Zhang et al., 2016; Hashem et al., 2017). Kim et al. reported that andrographolide inhibits LPS-induced macrophage inflammation by activating AMPK and suppressing NF-κB (Kim et al., 2019). Zhou et al. reported that berberine attenuates adjuvant-induced arthritis by promoting macrophage polarization to the M2 phenotype through the AMPK/NF-κB pathway (Zhou et al., 2019). In this study, our results revealed that *Il12a* deletion inhibited AMPK and promoted NF-κB activation. In addition, AMPK activation abolishes the deterioration effect of *Il12a* deletion on LPS-induced cardiac dysfunction. Moreover, AMPK activation also abolishes *Il12a* deletion-mediated M1 macrophage polarization and decreases macrophage-secreted proinflammatory production *in vitro*. These results show that *Il12a* deletion aggravates the imbalance of M1 and M2 macrophages by inhibiting AMPK and promoting NF-κB activation in macrophages.

In conclusion, *Il12a* deletion in mice aggravates LPS-induced cardiac injury and cardiac dysfunction by exacerbating the imbalance of M1 and M2 macrophages. Our data provide evidence that *Il12a* may be an attractive target for sepsis-induced cardiac dysfunction.

## Data Availability

The raw data supporting the conclusions of this article will be made available by the authors, without undue reservation.
