# Supplementary figures and images for: Il12a Deletion Aggravates Sepsis-Induced Cardiac Dysfunction by Regulating Macrophage Polarization
Source: Front Pharmacol. 2021 Jul 2;12:632912. doi: 10.3389/fphar.2021.632912 (PMC8284189; doi:10.3389/fphar.2021.632912)

Figure 1

A

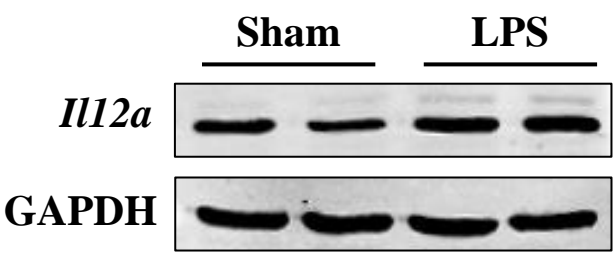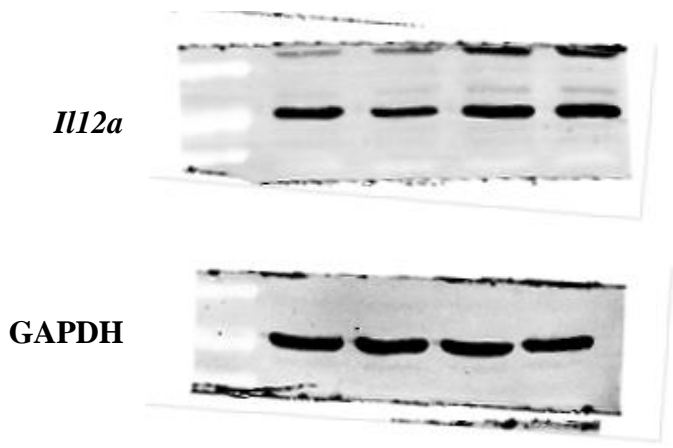

Figure 1

Figure 6

A

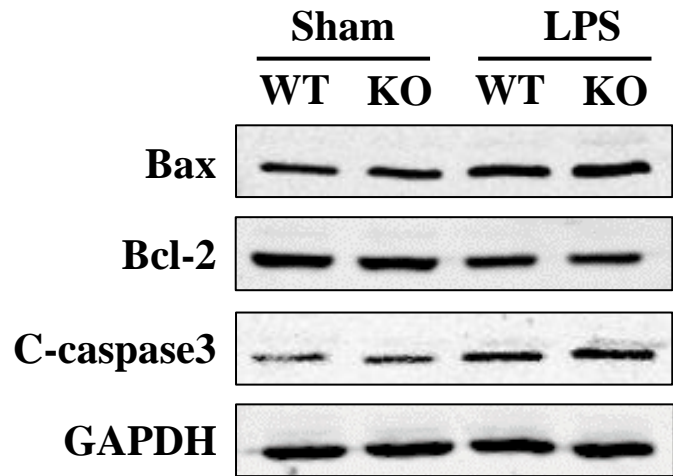

Bax

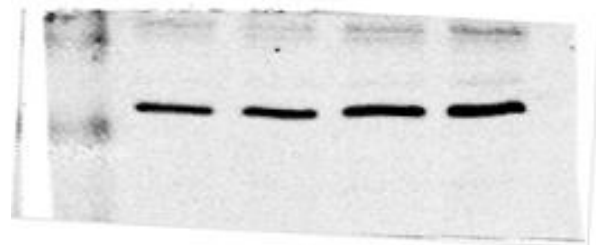

Bcl-2

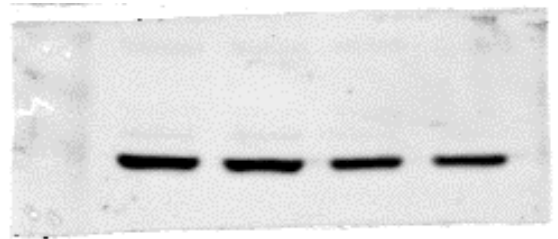

C-caspase3

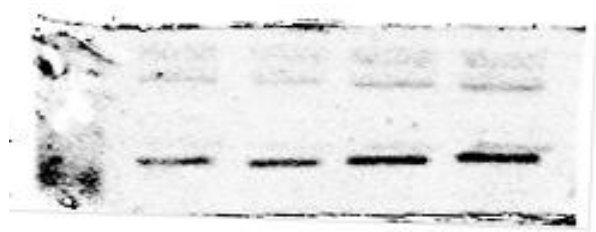

GAPDH

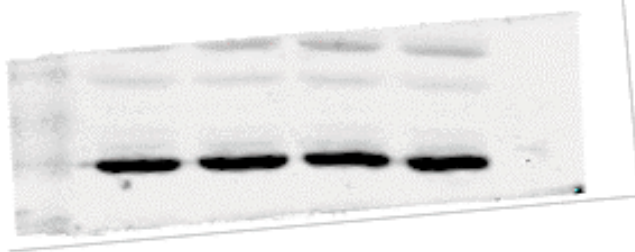

Figure 7

A

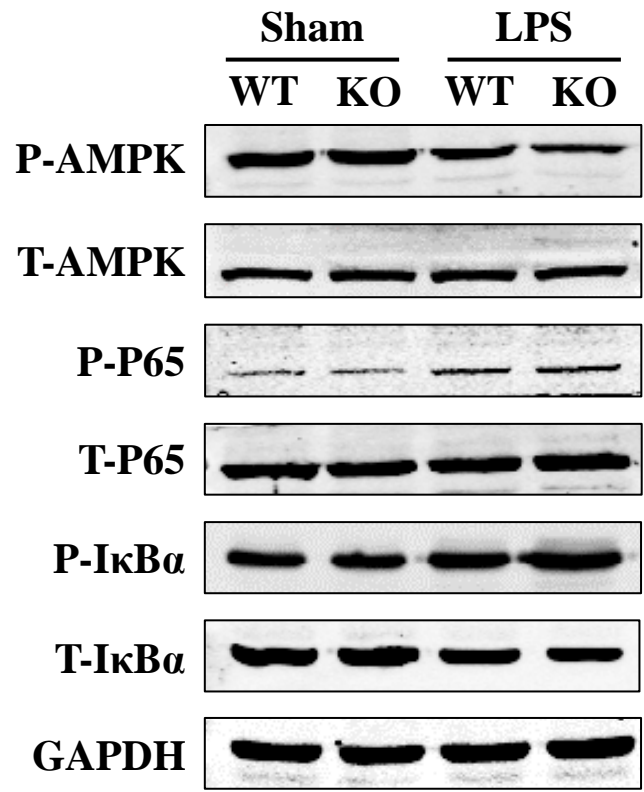

P-AMPK

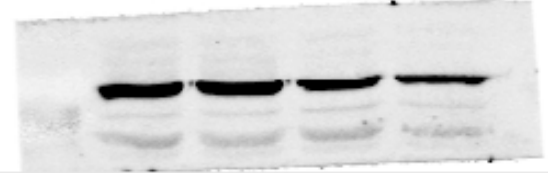

T-AMPK

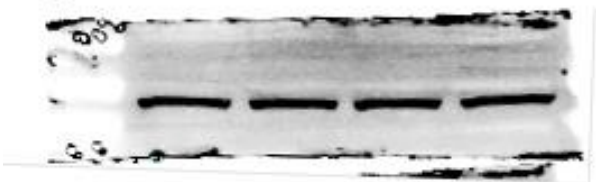

P-P65

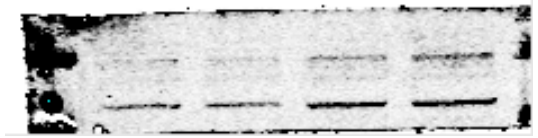

T-P65

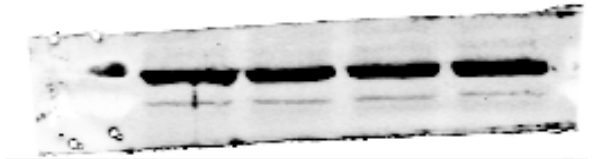

P-IκBα

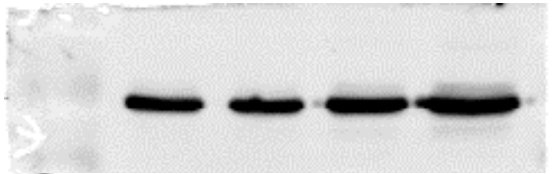

T-IκBα

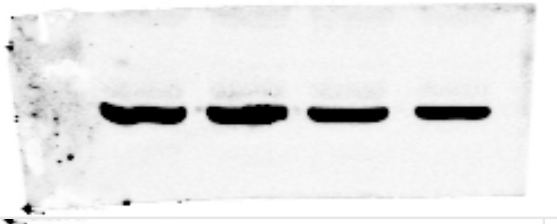

GAPDH

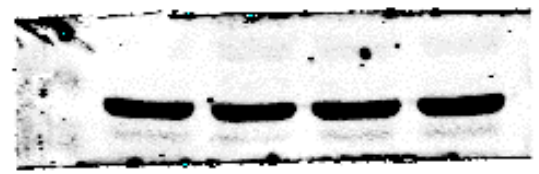

Supplement: Supplementary file 1 [file DataSheet1.pdf]
